# Medical Opinion and Movement

**Published:** 1907-04-13

**Authors:** 


### Medical Opinion and Movement.

The surgeon is frequently called upon to operate, with a view to relieving some condition which falls under the general heading of "the acute abdomen," without previously being able to arrive at a definite opinion as to the pathological lesion he is likely to find. In such cases, when there are no clear indications even of the region or viscus involved, it is usual to make an exploratory incision in the middle line below the umbilicus. Mr. Charles P. Childe, surgeon to the Royal Portsmouth Hospital, questions the advisability of this site for the incision, and advocates in preference the right semilunar line as the position of election. By a well-reasoned series of arguments and by a careful enumeration of all the possible causes which may give rise to the so-called "acute abdomen," he shows that the majority of these causes occur within an area of the abdomen which is most accessible and which can be most easily inspected by such an incision. As he points out, the success of the operation often depends upon a speedy recognition of the cause of the trouble, and for this purpose the choice of the site of incision is of the greatest importance. The usual median incision certainly gives the most ready access for the hand to all parts of the abdomen, but the incision he proposes is not far inferior in this respect, and it certainly appears to afford a better opening for the direct inspection of the greater number of lesions which are likely to underlie these conditions. Even with the experienced tactile skill of the surgeon, "an ounce of sight is worth a ton of touch."

Chloretone is a comparatively new drug, and is chiefly known as a sedative, both local and general. It is a white crystalline substance, only slightly soluble in water, but easily soluble in glycerine, alcohol, petroleum, and oil. It may be given in doses from 5 to 25 grains. It has been found useful in painful and irritable conditions of the stomach, and appears at the same time to retard fermentation. On this account it has been used to prevent sea-sickness, and has a considerable reputation in this respect. It is, in fact, regarded by some as a specific against the baneful effects of the motions of the ocean. Dr. W. Essex Wynter, of the Middlesex Hospital, has given considerable attention to the therapeutic value of this drug, and he has used it successfully in the treatment of chorea. He gives it in 5-grain doses in petroleum emulsion three times a day for two or three days, and then gradually reduces the dose. He has treated in all 14 cases, and has experienced uniformly good results. The treatment was continued for five to ten days, and in that time the chorin movements ceased, and the drug was replaced by tonics such as steel wine and arsenic or cod-liver oil. Even for mild cases this is a rapid recovery in comparison with what one usually expects under ordinary methods of treatment. But, according to Dr. Wynter, many of the cases were of a fairly severe type. It will be interesting to hear if a more general use of the drug confirms his results.

It is always difficult to devise measures of public utility which will effectually remedy some existing defect in the social cosmos without exerting a baneful influence in some other direction. The present high mortality of infants is undoubtedly a question of great moment, and one of national importance, and it is generally agreed that improper feeding is factor of the greatest significance in this respect. In order to provide the infants of the poor with a milk sterilised and properly prepared for their consumption, leading medical authorities have for some time urged the establishment of municipal milk depôts, and a Bill has lately been introduced into the House of Commons by the President of the Local Government Board to authorise local authorities to establish such depôts. Such a measure, by ensuring a supply of pure milk in a suitable condition for infant feeding, should materially conduce to a reduction in the heavy mortality among artificially fed infants. So far so good. What then of the possible evil results? In the first place, there is the tendency to lessen parental responsibility, which is inherent in all State or municipal measures, having as their object such care and provision of the children as naturally fall to the parents. But a point of still greater importance to consider is how far such measures will tend still further to reduce the proportion of breast-fed infants. When hand-feeding is made safer and easier, by the supply of milk ready prepared for the infant, will not the mother more readily yield to the temptation to give up suckling her infant?

This question has been ably discussed in an interesting paper by Dr. John F. J. Sykes, medical officer of health for St. Pancras. He emphasises the importance of influencing the health of the next generation through the health of the mother, and points out clearly that measures of reform should be directed to ensuring more favourable conditions for carrying out the maternal functions of pregnancy and lactation. As Dr. Sykes says, "In the hygiene of the expectant and suckling mother is contained the most powerful preventive of infantile mortality. If hand-feeding is encouraged, breast-feeding will be discouraged; if breast-feeding is discouraged, the incentive to maintain the health of the suckling mother will no longer remain; and if the prospect of suckling be removed, the preparation of the expectant condition of the mother will be neglected." Dr. Sykes makes many suggestions in regard to the limitation of the work of the mother both before and after confinement, and the provision for suckling the infant in cases of women employed in factories and workshops. He further proposes that some provision of supplemental feeding should be made for necessitous suckling mothers, guarded against abuse by the certificate of a woman inspector or voluntary visitor, and by a medical certificate as to health and breast-feeding. There can be no question that any measure which would encourage breast-feeding, and enable the mother to carry it out, would be of far greater utility in the prevention of infant mortality than any means that might be adopted to secure the most perfect form of bottle-feeding.

